# Convergent validity of the interRAI-HC for societal costs estimates in comparison with the RUD Lite instrument in community dwelling older adults

**DOI:** 10.1186/s12913-016-1702-1

**Published:** 2016-08-25

**Authors:** Lisanne I. van Lier, Henriëtte G. van der Roest, Hein P. J. van Hout, Liza van Eenoo, Anja Declercq, Vjenka Garms-Homolová, Graziano Onder, Harriet Finne-Soveri, Pálmi V. Jónsson, Cees M. P. M. Hertogh, Judith. E. Bosmans

**Affiliations:** 1 Department of General Practice and Elderly Care Medicine and EMGO+ Institute for Health and Care Research, VU University Medical Center, Van der Boechorststraat 7, 1081 BT Amsterdam, The Netherlands; 2 LUCAS, Centre for Care Research and Consultancy KU Leuven (University of Leuven), Minderbroederstraat 8, box 5310, B-3000 Leuven, Belgium; 3 HTW Berlin, FB III-Economics, Treskowallee 8, D-10318 Berlin, Germany; 4 Department of Geriatrics, Università Cattolica del Sacro, Largo F. Vito 1, 00168 Rome, Italy; 5 Department of Wellbeing, National Institute for Health and Wellbeing, P.O. BOX 30, FI-00271 Helsinki, Finland; 6 Department of Geriatrics, Landspitali National University Hospital and Faculty of Medicine, University of Iceland, Landakot, 101, Reykjavik, Iceland; 7 Department of Health Sciences and EMGO+ Institute of Health and Care Research, Faculty of Earth and Life Sciences, VU University, De Boelelaan 1085, 1081 HV Amsterdam, The Netherlands

**Keywords:** Convergent validity, Correlation, Resource utilisation, Costs of care, Societal costs, Routine care assessment, InterRAI-HC, RUD Lite, Community care, Older adults

## Abstract

**Background:**

The interRAI-Home Care (interRAI-HC) instrument is commonly used in routine care to assess care and service needs, resource utilisation and health outcomes of community dwelling home care clients. Potentially, the interRAI-HC can also be used to calculate societal costs in economic evaluations. The purpose of this study was to assess the convergent validity of the interRAI-HC instrument in comparison with the RUD Lite instrument for the calculation of societal costs among care-dependent community dwelling older adults.

**Methods:**

A within-subject design was used. Participants were 65 years and older and received professional community care in five countries. The RUD Lite was administered by trained (research) nurses or self-reports within 4 weeks after the interRAI-HC assessment. Agreement between the interRAI-HC and RUD Lite estimates was assessed using Spearman’s correlation coefficients. We hypothesised that there was strong correlation (Spearman’s *ρ* > 0.5) between resource utilisation estimates, costs of care estimates and total societal cost estimates derived from both instruments.

**Results:**

Strong correlation was found between RUD Lite and interRAI-HC resource utilisation assessments for eight out of ten resource utilisation items. Total societal costs according to the RUD Lite were statistically significantly lower than according to the interRAI-HC (mean difference €-804, 95 % CI −1340; −269). The correlation between the instruments for total societal costs and all six cost categories was strong.

**Conclusions:**

The interRAI-HC has good convergent validity as compared with the RUD-Lite instrument to estimate societal cost of resource utilisation in community dwelling older adults. Since interRAI-HC assessments are part of routine care in many community care organisations and countries already, this finding may increase the feasibility of performing economic evaluations among community dwelling older adults.

**Electronic supplementary material:**

The online version of this article (doi:10.1186/s12913-016-1702-1) contains supplementary material, which is available to authorized users.

## Background

The population in Europe is ageing rapidly [1]. Between 2010 and 2050, the proportion of adults aged 65 years and older is expected to increase from 8 to 16 % [2]. Many older adults experience difficulties in activities of daily living due to chronic illnesses or health-related disabilities which limit their ability to live independently in their homes which may be further complicated by cognitive problems [3]. However, most older adults want to continue to live independently in their own environment for as long as possible, and this is also encouraged by many European governments [2, 3]. As a consequence, the demand for (long-term) formal and informal care services is expected to grow substantially in the coming decades, which will put heavy pressure on health care systems across Europe [2, 4]. Since budgets available for health care are limited, policy makers need to make decisions on how to allocate health care resources in the most efficient way.

Economic evaluations can inform such allocation decisions by providing information on the relative efficiency of alternative health care interventions [5]. To estimate costs in economic evaluations, the utilisation of health care and social care resources needs to be quantified. Specific instruments have been developed to collect information on resource utilisation retrospectively by means of self-report structured questionnaires or interviews, such as the Resource Utilization in Dementia (RUD) Lite instrument [6]. Another way to collect this information from clients, is by using routine care assessments, that are administered by a health professional who is involved in the care for the client. When using routine care assessments, individuals are not exposed to additional questionnaires for measuring resource utilisation in economic evaluations which may be an important advantage in vulnerable patient groups such as care-dependent older adults. An example of a routine care instrument for this specific population is the interRAI Home Care instrument (interRAI-HC). The interRAI-HC is a standardised multidimensional geriatric assessment instrument that has been designed to assist in care planning, outcome measurement, quality improvement, and resource allocation for clients who receive care at home [7–9]. In the interRAI-HC, resource allocation is based on the advanced case-mix classification system “Resource Utilization Groups III Home Care (RUG-III-HC)” [10, 11]. The RUGs are based on client characteristics and do not reflect ‘actual’ care utilisation rates. Although the interRAI-HC was not specifically developed to estimate costs of resource utilisation, Brown et al. [12] have previously used this instrument to do this. However, when using the interRAI-HC to calculate costs of resource utilisation over a period of three months or longer, utilisation of health care services has to be extrapolated to longer periods. It is unclear whether this results in valid estimates of resource utilisation and costs over a period of three months or more. This is in contrast with the RUD Lite instrument which was specifically developed to measure utilisation of formal and informal care services and is widely used to estimate societal costs in community-dwelling people with dementia [6, 13, 14].

In order to evaluate whether the interRAI-HC can be validly used to estimate resource utilisation and associated costs, the convergent validity of the interRAI-HC instrument is studied in comparison with the RUD Lite instrument in a sample of care-dependent community dwelling older adults. Both resource utilisation of formal and informal care services and cost estimates between the two instruments will be compared.

## Methods

### Design

This study is part of the cross-European IBenC (“Identifying best practices for care-dependent elderly by Benchmarking Costs and outcomes of community care”) project that aims to provide insight into the costs and quality of community care delivery systems across Europe [15]. The study was approved by relevant legal authorised medical ethical committees in the countries that participated in the IBenC project (Belgium, Finland, Germany, Iceland, Italy, and The Netherlands).

For this sub-study, a within-subject design was used to evaluate the convergent validity of the interRAI-HC instrument in comparison with the RUD Lite instrument to measure resource utilisation and estimate costs from a societal perspective. Convergent validity was evaluated, since there is no gold standard for resource utilisation measurements. The data collection was conducted between January 2013 and March 2015.

### Setting and sample

Participants of the IBenC project were community dwelling adults aged 65 years and older who received care by a home care or community care organisation, or by a primary care nurse, and who were expected to receive care for at least six more months. In each participating country, one to six care organisations participated in the IBenC study. Per country, a subsample of at least 50 participants and their primary informal caregivers were selected for participation in this sub-study. Terminally ill persons and cognitively impaired persons (score of three or higher on the Cognitive Performance Scale (CPS) [16]) without an informal caregiver who was willing to participate as a proxy, were not included in the sub-study.

### Procedure

Clients receiving care from community care organisations that were involved in the IBenC project and who fulfilled the inclusion criteria were invited to participate, or automatically enrolled in the IBenC study in accordance with local ethical regulations. Prior to the start of the assessments, written informed consent was obtained from the participants. When a participant was known to be cognitively impaired (CPS ≥ 3 [16]), informed consent from a close relative, legal representative or legal guardian on behalf of the participant was obtained.

Two third of the participating community care organisations used the interRAI-HC instrument in routine care to monitor the health and care status of their clients. In community care organisations that did not use the interRAI-HC instrument in routine care, (research) nurses were trained to perform the interRAI-HC assessments. The assessments were completed based on observation from the (research) nurse, information from medical records, and information obtained by interviewing the client and their informal caregiver (if available). InterRAI-HC assessments were performed at baseline, after 6 and 12 months.

At the start of the data collection period, participants were invited by community care organisations to participate in an additional assessment with the RUD Lite (according to local protocols). The RUD Lite assessment took place at the home of the participant within 4 weeks after the index interRAI-HC assessment and was performed as an interview by a trained (research) nurse. A brief description of the aims of the IBenC study was provided during the training. If the participant was cognitively impaired, the (primary) informal caregiver completed the assessment. During the assessment, participants without cognitive impairment were asked for consent to contact their primary informal caregiver, in order to interview him or her on the amount of informal care provided to the participant. If participants did not consent, they answered these questions themselves. In Finland, the RUD Lite assessments were completed by participants or informal caregivers themselves by means of a written questionnaire. In case of difficulty due to, for example, cognitive impairment participants were assisted by a nurse (*n* = 18).

### InterRAI-HC instrument

In the interRAI-HC [7–9], information on the utilisation of home health care (home health aid), home nursing, homemaking services, physical therapy, occupational therapy, and psychological treatment, is collected by registering the number of days and the total number of minutes of care received in the 7 days prior to the assessment. With regard to physical therapy, occupational therapy, and psychological treatment, we assumed that the number of days per week the service was received, reflected the number of sessions received during a week. The utilisation of the supportive care service “meals on wheels” is registered in number of days the service was used during the 7 days prior to the assessment. The number of hospital admissions, emergency room visits and visits to a physician (specialist, authorised assistant or general practitioner) are registered over the 90 days prior to the assessment. The total number of hours of all informal care and active monitoring provided by informal carers to a participant are assessed in the 3 days prior to the assessment.

In order to estimate the amount of resource utilisation over a period of 3 months, resource utilisation items with a recall period of 7 days were extrapolated to reflect a period of 3 months. Resource utilisation estimates (number of days, hours of care, or number of sessions) were multiplied by 13 (3months correspond to 13 weeks). Informal care hours were divided by three and multiplied by 91. The interRAI-HC assesses the number of hospital stays but does not assess the number of nights. To estimate the number of nights, we used country-specific averages of length of stay during hospital admission in the year 2012 and multiplied these rates by the number of hospital admissions (see Table 1 [17]).

**Table 1:** Overview of used unit cost (in € 2015) and average length of stay (days)

Care service	Costs (€) per unit
Home care	
Home health and domestic care (including home health care, home nursing and home making services)	38.00 per hour
Physician visits	
General practitioner visit	30.40 per visit
Outpatient clinic visits	78.16 per visit
Other health care services	
Physical therapy	39.08 per session
Occupational therapy	23.88 per session
Psychological treatment	86.85 per session
Hospital admissions	
Hospital admission with overnight stay	
General ward	496.11 per day with overnight stay
ICU	2369.82 per day with overnight stay
Average length of hospital stay^a^	
Belgium	6.7 days
Finland	11.0 days
Germany	9.2 days
Iceland	5.8 days
Italy	7.7 days
The Netherlands	5.2 days
Emergency room visit (without overnight stay)	163.92 per visit
Supportive care services	
Meals on wheels	7.06 per day
Informal care	
Informal care	13.57 per hour

^a^Source: OECD, 2015

The interRAI-HC includes several functional scales which were used to describe the study population. Cognitive functioning was assessed using the Cognitive Performance Scale (CPS, range 0–6). Moderate or severe cognitive impairment was considered to be present if CPS ≥ 3 [16]. The Depression Rating Scale (DRS, range 0–14) was used to assess depressive symptoms. A score of three or more on the DRS indicates minor or major depressive disorder [18]. Activities of daily living (ADL) needs were assessed using the interRAI Activities of Daily Living Hierarchy Scale (ADLH, range 0–6) with higher scores indicating higher ADL needs [19]. Difficulty in performing instrumental activities (iADL) was assessed using the interRAI Instrumental ADL Performance Scale (iADLP, range 0–48) with higher scores indicating more iADL dependencies [20]. Pain was considered to be present if the score on the Pain Scale (range 0–3) was one or higher [21]. Multimorbidity was defined to be present when an individual indicated to have two or more chronic medical conditions [22].

### RUD Lite

The RUD Lite was specifically developed to measure resource utilisation from a societal perspective among older adults with dementia [6, 13, 14, 23, 24]. Although the RUD Lite was originally developed to measure resource utilisation in people with dementia, the services that are covered by the instrument are also used by vulnerable community-dwelling older adults without dementia. The validity of the instrument has been studied extensively, especially the items that assess caregiver time [13, 24]. Therefore, we chose this instrument to compare resource utilisation according to the interRAI-HC with. Moreover, the RUD Lite (version 3.2) was available in five of the six languages of the countries that participated in the IBenC project.

The RUD instrument is divided into two subsections; a section that assesses background information of the client and his/her utilisation of health care services and a section that assesses caregiver time.

For this specific study, the recall period for all items in the questionnaire was extended from 30 days to 3 months to match up with interRAI-HC recall periods. The frequency of service use was changed from the number of visits during the last month into the average number of visits per week during the last 3 months. We made two versions, a client version, in which the questions were directly targeted at the clients, and a caregiver version. The latter was used when the caregiver answered the questions instead of the client. The adapted language versions were translated for use in the IBenC study by a process of forward translation, reconciliation and back translation review. The translations were performed by independent qualified translators.

Utilisation of home health care, home nursing, and homemaking services, was registered as average number of times per week and average number of hours and minutes per visit the service was received in the 3 months prior to the assessment. Use of meals on wheels was recorded as average number of service meals received per week. Physical therapy, occupational therapy, psychological treatments, emergency room visits, general practitioner visits and outpatient clinic visits were registered as total number of visits in the 3 months prior to the assessment. The number of hospital admissions and the total length of stay (number of nights) stratified by ward type (general ward and Intensive Care Unit (ICU)) in the past 3 months was recorded. Informal care provision (personal ADL and instrumental ADL) and supervision (or surveillance) provided by the primary informal caregiver was assessed as total number of days during the last 3 months, as well as the number of hours and minutes on a typical care day during this period.

In order to estimate the amount of resource utilisation over a period of 3 months, the average use of care services recorded on a weekly basis was extrapolated by multiplying the estimates by 13. The number of days on which informal care was provided was multiplied by the recorded number of hours of informal care received. Also, the share of care provisioning by the primary informal caregiver was recorded (1–20 %, 21–40 %, 41–60 %, 61–80 %, 81–100 %) and the number of other informal caregivers involved. The total amount of time of informal care was estimated by dividing the median value of the answer categories by the amount of caregiving time.

### Cost estimates

Standardised costs were used for all countries in order to avoid variations in costs due to country specific differences in care valuation. Since European standard costs are lacking, resource utilisation was valued using Dutch standard costs [25]. Because of country specific categorisation of health care and social service provisioning, it was not possible to make a clear distinction between the utilisation of different types of home care services. Therefore, hours of home health care, home nursing and homemaking services were first summed into “home health and domestic care”, and then valued using the weighted standard cost for home care [25]. All costs were adjusted to the year 2015 using consumer price indices [26]. Six cost categories were distinguished: home health and domestic care, physician visits, other health care services, hospital admissions, supportive care services, and informal care. Additionally, these cost categories were summed into total societal costs. Table 1 lists the care services per cost category and prices per unit as used in this study. With regard to physician visits, in contrast to the RUD Lite, the interRAI-HC makes no distinction between outpatient clinic visits and general practitioner visits. We assumed that most visits were visits to an outpatient clinic. Therefore, physician visits assessed with the interRAI HC were valued using the price of outpatient clinic visits.

### Statistical analysis

All analyses were performed using SPSS statistics 20 [27]. Demographic and clinical characteristics of the participants, utilisation of formal and informal care, and costs estimates were described using descriptive statistics and frequencies.

Differences in baseline characteristics between participants from different countries were evaluated using Chi-square tests for categorical variables and ANOVAs for continuous variables.

Mean differences in utilisation rates and costs between the RUD Lite and interRAI-HC were statistically tested using paired sample t-tests. Because of the skewed distribution of the resource utilisation and cost data, 95 % confidence intervals (CIs) were estimated using bias-corrected accelerated bootstrapping (5000 replications) [28].

The agreement between the resource utilisation measurements and cost estimates of the interRAI-HC instrument and the RUD Lite instrument was assessed using Spearman’s ρ correlation coefficients, since the distribution of resource utilisation and costs were skewed. According to Cohen et al. correlation of 0.10–0.30 corresponds to weak correlation, 0.30–0.50 to moderate correlation and 0.50 or higher corresponds to strong correlation [29].

To evaluate the convergent validity of the interRAI-HC for resource utilisation measurement as compared to the RUD Lite, we hypothesised that the strength of the correlation between interRAI-HC and the RUD Lite *resource utilisation items* was strong (Spearman’s *ρ* > 0.50). Ten predefined hypotheses on resource utilisation were tested: hours of home health and domestic care, number of physician visits, number of physical therapy sessions, number of occupational therapy sessions, number of psychological treatment sessions, number and duration of hospital admissions, number of emergency room visits, number of meals on wheels, and hours of informal care. We also hypothesised that the correlation between *cost of care estimates* within the six cost categories and the total societal cost of resource utilisation collected with the interRAI-HC and the RUD Lite was strong (seven hypotheses, Spearman’s *ρ* > 0.50). In total 17 hypotheses were tested.

The correlation between the total societal costs of resource utilisation according to the two instruments were also analysed using a Bland-Altman plot [30]. For each participant, the mean of the total societal costs based on the RUD Lite and the interRAI-HC was plotted against the difference in mean total societal costs between the RUD Lite and the interRAI-HC. The variability of the differences in total societal cost estimates between the two instruments and the limits of agreement, calculated as mean difference +/- 1.96 SD, were visualized in this plot. The limits of agreement can be interpreted as the interval in which approximately 95 % of the differences in total societal cost estimates between the two instruments should lie. The smaller the range between these two limits, the better the agreement between both instruments is.

Participants from Italy (*n* = 102) were excluded from data-analyses due to protocol violation; the resource utilisation section of the RUD Lite was completed with data derived from the client’s administrative chart which also formed the basis for the interRAI-HC assessment. Furthermore, participants from Belgium (*n* = 103) were also excluded from the main analysis since the amount of caregiving time was not assessed. This item was not available in the Belgian interRAI-HC software.

### Sensitivity analysis

A sensitivity analysis was performed to test the correlation between cost of care estimates from a health care perspective, meaning that informal care costs were excluded from the analysis. We hypothesised that the correlation between total health care cost estimates with the interRAI-HC and the RUD Lite was strong as well (Spearman’s *ρ* > 0.50). In this sensitivity analysis, Belgian participants were included.

The interRAI-HC does not collect information on length of hospital stay. Therefore, country-specific averages of length of stay (in days) during hospital admission of the year 2012 based on the OECD database were used to estimate the number of nights spend in the hospital based on the number of hospital admissions as collected with the interRAI-HC. We did a sensitivity analysis in which we subtracted 1 from the average number of hospital days to obtain an estimate of the average number of hospitalisation nights.

Furthermore, a sensitivity analysis was performed to assess the correlation between both instruments stratified for people with cognitive problems (CPS ≥3) and people without cognitive problems.

Different administration modes of the RUD Lite questionnaire were used in this study; the RUD Lite was administered as an interview with the client, with the client and caregiver together or with the caregiver alone. Also, in Finland the RUD Lite questionnaires were completed on paper by the client or caregiver themselves. In a few cases, nurses from the care agency assisted the client completing the questionnaire. A sensitivity analysis was performed to evaluate the effect of the different administration modes of the RUD Lite on the correlation between the RUD Lite and the interRAI-HC.

## Results

### Study sample

The subsample consisted of 790 participants. In total, 134 (17 %) subjects were excluded from the main analysis due to missing values on one or more resource utilisation items: 103 from Belgium, three from Germany, 23 from Finland, and five from the Netherlands. Compared to the participants, the excluded subjects were statistically significantly (*p* < 0.05) younger, suffered relatively more often from cognitive impairment and depression, scored higher on ADL and iADL, experienced multimorbidity less frequently, and had a higher number of caregivers.

In total, 656 (83 %) participants were included in the analyses. Participants were on average 83.2 years of age (SD 7.2), 67 % was female and 24 % of the participants was dependent in at least one of four ADLs (personal hygiene, toilet transfer, locomotion and/or eating (score of two or higher on ADLH)).

Statistically significant differences (*p* < 0.05) between participants across countries were found for age, living status, CPS, DRS, ADL, iADL, pain, multimorbidity and number of caregivers (see Table 2).

**Table 2:** Characteristics of the study population

	Total (*n* = 656)	Finland (*n* = 346)	Germany (*n* = 60)	Iceland (*n* = 103)	Netherlands (*n* = 147)	Test statistics	*p*-value
Mean age (SD)	83.2 (7.2)	83 (7.2)	84.1 (8.0)	84.7 (6.2)	82.2 (7.2)	*F* = 2.97	0.03
Female (n, %)	439 (67 %)	231 (67 %)	38 (63 %)	71 (69 %)	99 (67 %)	*χ* ^*2*^ = 0.55	0.91
Living alone (n, %)	472 (72 %)	277 (80 %)	36 (60 %)	65 (63 %)	94 (64 %)	*χ* ^*2*^ = 24.17	<0.01
Cognitive impairment (CPS ≥ 3) (n, %)	61 (9 %)	40 (12 %)	13 (22 %)	6 (6 %)	2 (1 %)	*χ* ^*2*^ = 25.44	<0.01
Depressive symptoms (DRS ≥ 3) (n, %)	82 (13 %)	25 (7 %)	7 (12 %)	12 (12 %)	38 (26 %)	*χ* ^*2*^ = 32.86	<0.01
Mean ADLH score (SD)	0.8 (1.4)	0.7 (1.3)	2.4 (1.9)	0.5 (0.9)	0.7 (1.4)	*F* = 31.55	<0.01
Mean iADLH score (SD)	25.4 (12.8)	27.3 (12.6)	27.8 (16.3)	23.5 (11.1)	21.3 (11.8)	*F* = 8.52	<0.01
Pain (Pain Scale > 0) (n, %)	392 (60 %)	226 (66 %)	19 (32 %)	69 (67 %)	78 (53 %)	*χ* ^*2*^ = 29.75	<0.01
Multimorbidity (n, %)	376 (57 %)	208 (60 %)	23 (38 %)	63 (61 %)	82 (56 %)	*χ* ^*2*^ = 10.71	0.01
Having an informal caregiver (n, %)						*χ* ^*2*^ = 137.15	<0.01
No caregiver present	81 (12 %)	58 (17 %)	6 (10 %)	0 (0 %)	17 (12 %)		
One caregiver	235 (36 %)	157 (45 %)	37 (62 %)	3 (3 %)	38 (26 %)		
Two or more caregivers	340 (52 %)	131 (38 %)	17 (28 %)	100 (97 %)	92 (63 %)		

In 21 % of the cases, the RUD Lite was administered as an interview with the participant, another 18 % with the participant and caregiver together, and 9 % with the caregiver alone. In Finland, paper versions of the RUD Lite were completed by the participant (16 %), the participant and the caregiver together (26 %) or by caregiver themselves (7 %). In 18 cases (3 %), Finnish nurses from the care agency assisted the participant completing the questionnaire.

### Resource utilisation

Table 3 provides an overview of the utilisation rates of formal and informal care services over a period of 3 months as assessed with the RUD Lite and the interRAI-HC. Resource utilisation as assessed with the RUD Lite was significantly higher for number of physician visits, and significantly lower for number of hours of home health and domestic care services received, duration of hospital admissions and number of meals as compared to interRAI-HC assessments. All other differences in resource utilisation estimates between the RUD Lite and interRAI-HC were not statistically significant.

**Table 3:** Resource utilisation over a three month period assessed with the RUD Lite and InterRAI-HC

	RUD Lite (*n* = 656)		InterRAI-HC (*n* = 656)		Mean Difference (RUD Lite minus interRAI-HC)	Spearman’s ρ (range countries)
*Service use category*	Use of service, n (%)	Mean (SD)	Use of service, n (%)	Mean (SD)	Mean (95 % CI)	
Home care						
Home health and domestic care hours	544 (83 %)	62.4 (75.3)	641 (98 %)	68.5 (65.9)	−6.1 ( −10.9; −1.4)	0.56* (0.38*- 0.81*)
Physician visits						
Physician visits (GP + outpatient clinic visits	385 (59 %)	1.7 (2.6)	275 (42 %)	1.2 (2.3)	0.5 (0.3; 0.6)	0.62* (0.03–0.75*)
General practitioner visits	296 (45 %)	1.0 (1.6)	-	-	-	-
Outpatient clinic visits	189 (29 %)	0.7 (1.8)	-	-	-	-
Other health care services						
Physical therapy sessions	137 (21 %)	2.6 (6.6)	89 (14 %)	2.6 (7.2)	0.1 (−0.3; 0.5)	0.68* (0.27*–0.82*)
Occupational therapy sessions	24 (4 %)	0.2 (1.1)	15 (2 %)	0.5 (3.8)	−0.3 (−0.7;−0.1)	0.14* (−0.01–1.00*)
Psychological treatment	5 (1 %)	0.0 (0.2)	4 (1 %)	0.1 (1.1)	−0.1 (−0.1;−0.0)	0.67* (0.00-0.86*)
Hospital admissions						
Hospital admission with overnight stay, times	93 (14 %)	0.2 (0.6)	99 (15 %)	0.3 (1.1)	−0.1 (−0.2; 0.0)	0.57* (0.13–1.00*)
Hospital admission with overnight stay, nights	86 (13 %)	1.2 (4.9)	99 (15 %)	2.3 (7.5)^a^	−1.1 (−1.7;−0.6)	0.54* (0.14–1.00*)
Nights general ward	84 (13 %)	1.2 (4.8)	-	-	-	-
Nights ICU	4 (1 %)	0 (0.2)	-	-	-	-
Emergency room visits without overnight stay	92 (14 %)	0.2 (1.1)	87 (13 %)	0.2 (0.7)	0.0 (0.0; 0.1)	0.35* (0.00–0.74*)
Supportive care services						
Meals on wheels	235 (36 %)	25.1 (39.2)	279 (43 %)	31.4 (39.5)	−6.3 (−8.7;−4)	0.72* (0.59*–0.92*)
Informal care						
Informal caregiver time	413 (63 %)	212 (498.9)	483 (74 %)	211.2 (426.4)	0.8 (−28.1; 31.6)	0.61* (0.48*–0.80*)

* *p* < 0.01

^a^ Estimated using OECD data [17]

Table 3 also shows that eight out of 10 predefined hypotheses regarding the correlation between RUD Lite and interRAI-HC resource utilisation measurement were confirmed (number of hours of home health and domestic care services received, number of physician visits, physical therapy sessions and psychological treatment sessions, the number and duration of hospital admissions, the number of meals, and the amount of informal caregiver time). For the number of occupational therapy sessions and emergency room visits, our hypotheses could not be confirmed (Spearman’s *ρ* < 0.5). Country-specific resource utilisation estimates and the correlation between the two types of assessments can be found in Additional file 1.

In short, for Iceland, we found moderate correlation (0.3 < Spearman’s *ρ* < 0.5) for the number of emergency room visits and physician visits, and strong correlation for all other resource utilisation services (Spearman’s *ρ* > 0.5). The results from Germany showed strong correlation for the number of physician visits, the number of meals and the amount of informal caregiver time, and moderate to weak correlation for the other resource utilisation services (0.1 < Spearman’s *ρ* < 0.5). For Finland, strong correlation was found for two services, including physical therapy sessions, and number of meals. For the Netherlands, weak correlation (0.1 < Spearman’s *ρ* < 0.3) was found for the number of occupational therapy sessions and strong correlation was found for all other resource utilisation services.

### Costs of care

Table 4 provides an overview of the estimated costs over a period of 3 months as assessed with the RUD Lite and the interRAI-HC. Estimated costs assessed with RUD Lite as compared to interRAI-HC assessments were significantly lower for home health and domestic care (mean difference €-233, 95 % CI −415; −54), hospital admissions (mean difference €-517, 95 % CI −786; −246), supportive care services (mean difference €-45, 95 % CI −61; −29), and total societal costs (mean difference €-804, 95 % CI −1340; −269). The differences in other cost categories between RUD Lite and interRAI-HC assessments were not significant.

**Table 4:** Cost estimates (€) over a three month period assessed with the RUD Lite and interRAI-HC

	RUD Lite (*n* = 656)	InterRAI-HC (*n* = 656)	Mean Difference (RUD Lite minus interRAI-HC)	Spearman’s ρ (range countries)
*Service use category*	Mean (SD)	Mean (SD)	Mean (95 % CI)	
Home health and domestic care	2369 (2860)	2603 (2505)	−233 (−415; −54)	0.56* (0.37*–0.81*)
Physician visits	83 (158)	93 (178)	−10 (−21; 2)	0.57* (0.06–0.72*)
Other health care services	108 (265)	118 (316)	−10 (−29; 8)	0.66* (0.35*–0.79*)
Hospital admissions	680 (2568)	1197 (3737)	−517 (−786; −246)	0.53* (0.14–0.91*)
Supportive care services	177 (277)	222 (279)	−45 (−61; −29)	0.72* (0.59*–0.92*)
Informal care	2877 (6770)	2866 (5787)	10 (−382; 428)	0.61* (0.48*–0.80*)
Total societal costs	6295 (8221)	7099 (7428)	−804 (−1340; -269)	0.60* (0.51*–0.79*)

* *p* < 0.01

All seven predefined hypotheses on the correlation of the cost of care estimates between the RUD Lite and interRAI-HC were confirmed (Spearman’s *ρ* > 0.5). Country-specific cost estimates and correlation between the RUD Lite and interRAI-HC assessment can be found in Additional file 2. The results of Iceland showed strong correlation between the RUD Lite and interRAI-HC for all cost categories and total societal costs, except for costs of physician visits (Spearman’s *ρ* < 0.5). Strong correlation was also found for the estimated costs of supportive care services, informal care, and total societal costs in Germany, and for the estimated costs of supportive care services and total societal costs in Finland. For the Netherlands, strong correlation was found for all costs categories and total societal costs estimates between both instruments.

**Fig. 1:**
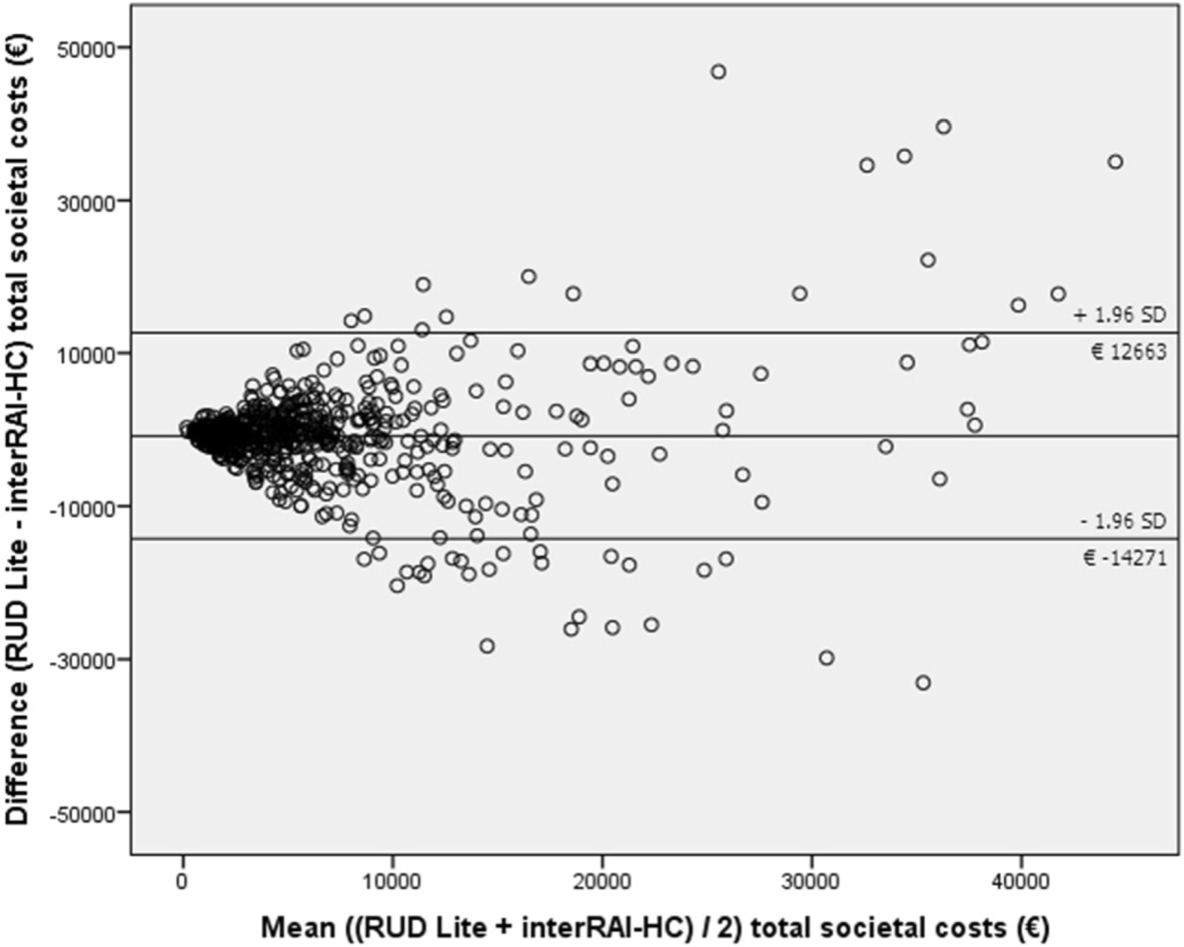
Bland and Altman plot comparing differences in costs assessed with the RUD Lite and interRAI-HC

The Bland-Altman plot shows that cost differences for the total societal costs between the two methods are becoming larger as the mean of the cost estimates based on the two methods is increasing (see Fig. 1). This becomes especially clear for participants for whom the mean of the two methods is €10000 or more. The 95 % limits of agreement are wide (−14271; 12663), showing considerable variation between the two methods of cost estimation.

### Sensitivity analysis

A total of 691 subjects were included in the analysis in which the correlation between cost of care estimates from a health care perspective was tested. The difference in total health care costs was €-685 between the RUD Lite and interRAI-HC (95 % CI -1007; −375). The correlation between the instruments for total health care costs was strong (Spearman’s ρ = 0.58).

The use of country-specific averages of length of stay during hospital admission based on the OECD database minus one day resulted in a smaller difference in total societal costs between the RUD Lite and the interRAI cost estimates (mean difference €-345 instead of €-804), but this difference was still statistically significant (95 % CI −590; −106).

After stratification for cognitive impairment, the difference in total societal costs was €-1288 between the RUD Lite and interRAI-HC resource utilisation assessment for people with cognitive impairment (95 % CI −3609; 1321), and €-755 for people without cognitive impairment (95 % CI −1256; −220). Strong correlation was found for total societal costs estimates in both people with cognitive impairment (Spearman’s ρ = 0.52), and people without cognitive impairment (Spearman’s ρ = 0.59).

The difference in total societal cost was €-1231 between the RUD Lite and interRAI-HC when the RUD Lite was administered as an interview with exclusively the participant (95 % CI −1890; −628). Relatively smaller mean differences in total societal costs estimates between the RUD Lite and interRAI-HC were found when the participant and the caregiver were interviewed together (mean difference €349, 95 % CI −1419; 2183) or when exclusively the caregiver was interviewed (mean difference €-204, 95 % CI −1957; 1524). When exclusively the caregiver was interviewed, a slightly adapted version of the RUD Lite was used, in which the questions were targeted at the caregiver instead of at the client. The caregiver answered the questions regarding care utilisation on behalf of the client.

When paper versions of the RUD Lite were used (Finland), the difference in total societal costs between the RUD Lite and interRAI-HC was €-2660 when the participant completed a paper version of the RUD Lite him/herself (95 % CI −4522; −1214); €-486 when the participant and the caregiver completed the RUD Lite together (95 % CI −2137; 1325); €-238 when the RUD Lite was completed by the caregiver alone (95 % CI −1684; 1286), and €-111 when the participant received help from a nurse when completing the questionnaire (95 % CI −809; 617).

Moderate correlation between the RUD Lite and interRAI-HC for total societal cost estimates was found when the RUD Lite was completed on paper by the participant with or without help from a nurse (Spearman’s rho = 0.43), and strong correlation was found for all other administration modes (Spearman’s rho = 0.53 - 0.72).

## Discussion

### Main findings

The objective of this study was to evaluate the convergent validity of the interRAI-HC instrument in comparison with the RUD Lite instrument for estimating resource utilisation and associated costs in community-dwelling care-dependent older adults from five European countries. In total, 15 of the 17 predefined hypotheses (88 %) were confirmed: strong correlation was found between *resource utilisation assessments* with RUD Lite and interRAI-HC for eight out of ten hypotheses (home health and domestic care, physician visits, physical therapy, psychological treatment, hospital admissions (number and duration), meals on wheels, and informal caregiver time), and all seven *cost of resource utilisation* hypotheses (home health and domestic care, physician visits, other health care services, hospital admissions, supportive care services, informal care and total societal care costs). The hypotheses for the number of occupational therapy sessions and emergency room visits could not be confirmed.

For the purpose of the study, only information on utilisation of formal and informal care services that was included in both questionnaires was taken into account. These care services cover health care services that are most frequently used by older adults in the community [31]. However, the interRAI-HC also includes a wide range of irregularly provided care services and preventive examinations. Most of these services are provided for specific diseases (e.g., chemotherapy) or very infrequently (e.g., mammography), as shown by Brown et al. [12] These services and examinations are expected to contribute only marginally to the total societal costs in a community dwelling population of older adults, and were, therefore, not included in this study [12]. On the other hand, the RUD Lite assesses cost categories that are not included in the interRAI-HC. These include care related transportation, psychiatrist, social worker and hours of day care received. Future research is needed to assess the contribution of these items to the total societal cost estimates and, subsequently, the necessity to include these additional items in the interRAI-HC to make the instrument more suitable for cost of care assessments.

The resource utilisation services included in this study are similar to other cost studies among older adults [32, 33]. Metzelthin et al. calculated the cost of care utilisation over a 24 month period for 346 community dwelling frail older adults in the Netherlands [33]. In that study, information on resource utilisation was collected from health care insurance registries, local hospitals, and directly from the respondents by means of telephone interviews and postal questionnaires. The cost estimates reported (over a 3 month period), were in line with the estimates found in our study. Only for home health and domestic care, hospital care, and informal care, approximately 150 to 400 % higher costs were found in the current study.

### Strengths and limitations

One of the strengths of this study is that participants from four Western European countries were included in the main analysis, making the results generalisable to various care contexts. Although some country differences in the correlation between the RUD Lite and the interRAI were present (See Additional files 1 and 2), the study results show good convergent validity of the interRAI-HC for resource utilisation measurement and costs of care estimates across countries. Another strength is that the interRAI-HC assessments were in most care organisations part of routine care. This has kept the burden for participants low. Additionally, costs were assessed from a societal perspective which is generally recommended by national guidelines [34]. The RUD Lite was chosen as reference instrument because previous studies showed that it has good clinimetric properties when assessing costs of resource utilisation of formal and informal care services [13, 24]. However, the RUD Lite cannot be considered a gold standard for measuring resource utilisation of formal and informal care services because it relies on self-report. Also, since the interRAI used a recall period of 3 months for some service utilisation items, the recall period of the RUD Lite was extended from 30 days to 3 months. Although it is suggested in the literature that recall periods up to 6 months can be reliably used to measure resource utilisation [35], it is unclear to what extent the validity of the RUD Lite is affected by this adaptation. This can be considered a potential limitation of the study.

Another limitation of the study is that we had to exclude approximately one sixth of the subjects from analyses due to missing values on the resource utilisation items in the interRAI-HC or RUD Lite. Also, cognitively impaired persons (CPS ≥ 3 [16]) without an informal caregiver who was willing to participate as a proxy, were not included in this study. This may affect the generalisability of the results. Significant differences were found in most of the demographic and clinical characteristics between the participants and the excluded subjects. Furthermore, the utilisation of some health care services, such as occupational therapy and psychological treatment was very low in some countries (1 % of the study population used this service on average). Therefore, the results found for these services should be interpreted with caution.

A number of assumptions was made in this study. Although hospital admissions are known to be a major cost driver for total health care costs, the interRAI-HC does not record the number of nights spent in a hospital. Therefore, we used the average number of hospitalisation days according to the OECD to calculate the total number of days a participant was admitted to a hospital. The OECD database provides internationally comparable statistics on a wide range of topics. We included data from the year 2012 as this was the most recent year for which complete data for all countries that participated in the IBenC project was available. Secondly, the interRAI-HC does not distinguish between general practitioner visits and outpatient clinic visits. A pragmatic choice was made to value physician visits with the price of outpatient clinic visits, since we assumed that most visits were to an outpatient clinic. However, this might have led to an overestimation of the costs for physician visits in the interRAI-HC. Another limitation concerns variation in assessor, mode and timing of the administration of the RUD Lite and the interRAI-HC across countries: two countries administered the instruments by the same assessor during the same contact or after a short period of time (Netherlands, Belgium), while in another country the assessors differed (Iceland) and the period was longer (Germany) or self-report instead of interview took place (Finland). In situations where the instruments were administered on the same day by the same assessor, the correlation between both instruments may be overestimated as compared to situations in which the assessor differed or the assessments took place on different days. Subsequently, we explored the effect of mode of the administration of the RUD-Lite on the correlation between both instruments and found lower correlation when the RUD Lite was completed by the participants themselves on paper instead as an interview. The use of self-report in Finland may thus potentially explain the weak to moderate correlation found for most resource utilisation services in this country.

## Conclusions

To the best of our knowledge, this is the first study to assess the convergent validity for societal cost of resource utilisation of an instrument that can be used in routine care, the interRAI-HC, as compared to a specifically developed resource utilisation instrument, the RUD Lite. The results show that the interRAI-HC has good convergent validity to estimate societal costs in community dwelling older adults. Next to the benefits of using the interRAI-HC as comprehensive geriatric assessment instrument, such as improved care planning, possibility to benchmark quality of care and efficiently allocate resources, this finding shows that the interRAI-HC can be used to estimate costs for use in economic evaluations thereby substantially improving the feasibility of performing economic evaluations among community dwelling older adults. Since the interRAI-HC is globally widely used in routine care in many organisations, the information is readily available and additional patient burden for the purpose of cost of care assessments can be avoided. However, to make the interRAI-HC more suitable for costs of care assessments, it is recommended to add the number of overnight hospital stays to the instrument, as well as to make a distinction between admission days on a general ward and an ICU and between visits to a general practitioner and a specialist. These adaptations are expected to result in more accurate cost estimates. Also, it is recommended to assess the influence of using country-specific valuations on the correlation between the cost of care estimates.

## Additional files

Additional file 1: Country specific resource utilisation. Resource utilisation estimates over a three month period assessed with the RUD Lite and InterRAI-HC by country (DOCX 44 kb) Additional file 2: Country specific cost of care estimates. Comparison of cost of care estimates (€) over a three month period between care utilisation assessed with the RUD Lite and interRAI-HC by country. (DOCX 28 kb)
