# Supplementary material for: Convergent validity of the interRAI-HC for societal costs estimates in comparison with the RUD Lite instrument in community dwelling older adults
Source: BMC Health Serv Res. 2016 Aug 25;16(1):440. doi: 10.1186/s12913-016-1702-1 (PMC5000456; doi:10.1186/s12913-016-1702-1)
Supplement: Additional file 1: — Country specific resource utilisation. Resource utilisation estimates over a three month period assessed with the RUD Lite and InterRAI-HC by country (DOCX 44 kb) [file 12913_2016_1702_MOESM1_ESM.docx]

**Additional file 1**. Country specific resource utilisation

Community care resource utilisation estimates over a three month period assessed with the RUD Lite and InterRAI-HC by country.

**Belgium**

|  | | **RUD Lite**  (n=32) | | **InterRAI-HC**  (n=32) | | **Mean Difference**  **(RUD Lite minus interRAI-HC)** | **Spearman’s ρ** |
| --- | --- | --- | --- | --- | --- | --- | --- |
| *Service use category* | | Use of ser-vice, n (%) | Mean (SD) | Use of ser-vice, n (%) | Mean (SD) | Mean (95% CI) |  |
| Home care | |  |  |  |  |  |  |
|  | Home health and domestic care hours | 31 (97%) | 171.7 (136.8) | 32 (100%) | 128.2 (96.3) | 43.5 (17.2; 76.1) | **0.81^*^** |
| Physician visits | |  |  |  |  |  |  |
|  | Physician visits (GP + outpatient clinic visits | 30 (94%) | 6.2 (7.0) | 18 (56%) | 2.6 (3.6) | 3.6 (1.5; 6.4) | 0.03 |
|  | General practitioner visits | 30 (94%) | 4.6 (3.3) | - | - | - | - |
|  | Outpatient clinic visits | 10 (31%) | 1.7 (6.4) | - | - | - | - |
| Other health care services | |  |  |  |  |  |  |
|  | Physical therapy sessions | 14 (44%) | 15.3 (20.8) | 10 (31%) | 14.2 (23.4) | 1.1 (-5.5; 7.6) | **0.56^*^** |
|  | Occupational therapy sessions | 10 (31%) | 3.6 (5.5) | 10 (31%) | 4.1 (6.1) | -0.5 (-1.1; -0.2) | **1.00*** |
|  | Psychological treatment | 0 (0%) | 0.0 (0.0) | 0 (0%) | 0.0 (0.0) | - | - |
| Hospital admissions | |  |  |  |  |  |  |
|  | Hospital admission with overnight stay, times | 4 (13%) | 0.2 (0.4) | 1 (3%) | 0.3 (1.4) | 0.0 (-0.6; 0.2) | **0.53*** |
|  | Hospital admission with overnight stay, nights | 4 (13%) | 1.9 (5.5) | 1 (3%) | 1.7 (9.5)** | 0.2 (-3.7; 3.3) | 0.42* |
|  | Nights general ward | 4 (13%) | 1.9 (5.5) | - | - | - | - |
|  | Nights ICU | 0 (0%) | 0.0 (0.0) | - | - | - | - |
|  | Emergency room visits without overnight stay | 3 (9%) | 0.1 (0.3) | 1 (3%) | 0.0 (0.2) | 0.0 (0.0; 0.2) | **0.56^*^** |
| Supportive care services | |  |  |  |  |  |  |
|  | Meals on wheels | 7 (22%) | 14.6 (30.2) | 6 (19%) | 13.8 (31.0) | 1.0 (-2,8; 6,0) | **0.92^*^** |
| Informal care | |  |  |  |  |  |  |
|  | Informal caregiver time | 25 (78%) | 1105 (2094) | - | - | - | - |

* p < 0.01

** Estimated using OECD data [17]

**Iceland**

|  | | **RUD Lite**  (n=103) | | **InterRAI-HC**  (n=103) | | **Mean Difference**  **(RUD Lite minus interRAI-HC)** | **Spearman’s ρ** |
| --- | --- | --- | --- | --- | --- | --- | --- |
| *Service use category* | | Use of ser-vice, n (%) | Mean (SD) | Use of ser-vice, n (%) | Mean (SD) | Mean (95% CI) |  |
| Home care | |  |  |  |  |  |  |
|  | Home health and domestic care hours | 103 (100%) | 44.4 (41.1) | 102 (99%) | 43.4 (42.5) | 1.1 (-2.4; 4.7) | **0.76*** |
| Physician visits | |  |  |  |  |  |  |
|  | Physician visits (GP + outpatient clinic visits | 72 (70%) | 1.5 (1.4) | 27 (26%) | 0.6 (1.2) | 0.9 (0.6; 1.2) | 0.38* |
|  | General practitioner visits | 34 (33%) | 0.4 (0.7) | - | - | - | - |
|  | Outpatient clinic visits | 59 (57%) | 1.1 (1.3) | - | - | - | - |
| Other health care services | |  |  |  |  |  |  |
|  | Physical therapy sessions | 31 (30%) | 5.2 (9.6) | 23 (22%) | 4.8 (10) | 0.4 (-1; 1.8) | **0.69*** |
|  | Occupational therapy sessions | 1 (1%) | 0.0 (0.1) | 0 (0%) | 0.0 (0.0) | 0 (0.0; 0.0) | 0 |
|  | Psychological treatment | 0.0 (0%) | 0.0 (0.0) | 0 (0%) | 0.0 (0.0) | - | - |
| Hospital admissions | |  |  |  |  |  |  |
|  | Hospital admission with overnight stay, times | 19 (18%) | 0.2 (0.5) | 18 (17%) | 0.2 (0.4) | 0.0 (0.0; 0.1) | **0.64*** |
|  | Hospital admission with overnight stay, nights | 19 (18%) | 1.7 (4.7) | 18 (17%) | 1.1 (2.4)** | 0.7 (0.0; 1.5) | **0.64*** |
|  | Nights general ward | 19 (18%) | 1.7 (4.7) | - | - | - | - |
|  | Nights ICU | 0 (0%) | 0 (0) | - | - | - | - |
|  | Emergency room visits without overnight stay | 24 (23%) | 0.3 (0.6) | 9 (9%) | 0.1 (0.5) | 0.2 (0.0; 0.3) | 0.30* |
| Supportive care services | |  |  |  |  |  |  |
|  | Meals on wheels | 25 (24%) | 14.9 (30.4) | 25 (24%) | 16.3 (31.5) | -1.4 (-6.1; 3.3) | **0.74*** |
| Informal care | |  |  |  |  |  |  |
|  | Informal caregiver time | 96 (93%) | 193.8 (269.4) | 92 (89%) | 229.1 (395.9) | -35.4 (-110.3; 27.5) | **0.80*** |

* p < 0.01

** Estimated using OECD data [17]

**Finland**

|  | | **RUD Lite**  n=346 | | **InterRAI-HC**  n=346 | | **Mean Difference**  **(RUD Lite minus interRAI-HC)** | **Spearman’s ρ** |
| --- | --- | --- | --- | --- | --- | --- | --- |
| *Service use category* | | Use of ser-vice, n (%) | Mean (SD) | Use of ser-vice, n (%) | Mean (SD) | Mean (95% CI) |  |
| Home care | |  |  |  |  |  |  |
|  | Home health and domestic care hours | 279 (81%) | 64.2 (85) | 343 (99%) | 71.5 (64.8) | -7.3 (-14.4; 0.6) | 0.48* |
| Physician visits | |  |  |  |  |  |  |
|  | Physician visits (GP + outpatient clinic visits | 128 (37%) | 0.8 (1.5) | 89 (26%) | 0.5 (1.2) | 0.3 (0.1; 0.4) | 0.38* |
|  | General practitioner visits | 101 (29%) | 0.6 (1.3) | - | - | - | - |
|  | Outpatient clinic visits | 44 (13%) | 0.2 (0.7) | - | - | - | - |
| Other health care services | |  |  |  |  |  |  |
|  | Physical therapy sessions | 34 (10%) | 0.5 (2.2) | 14 (4%) | 0.6 (2.8) | 0.0 (-0.3; 0.2) | 0.49* |
|  | Occupational therapy sessions | 2 (1%) | 0 (0.1) | 2 (1%) | 0.1 (1) | -0.1 (-0.2; 0.0) | -0.01 |
|  | Psychological treatment | 2 (1%) | 0 (0.1) | 0 (0%) | 0 (0) | 0.0 (0.0; 0.0) | 0 |
| Hospital admissions | |  |  |  |  |  |  |
|  | Hospital admission with overnight stay, times | 55 (16%) | 0.3 (0.7) | 64 (18%) | 0.3 (0.6) | 0.0 (-0.1; 0.1) | 0.48* |
|  | Hospital admission with overnight stay, nights | 48 (14%) | 1.4 (5.7) | 64 (18%) | 2.9 (7.1)** | -1.5 (-2.3; -0.8) | 0.45* |
|  | Nights general ward | 48 (14%) | 1.4 (5.6) | - | - | - | - |
|  | Nights ICU | 1 (0%) | 0.0 (0.2) | - | - | - | - |
|  | Emergency room visits without overnight stay | 55 (16%) | 0.3 (1.5) | 64 (18%) | 0.3 (0.9) | 0.0 (-0.1; 0.2) | 0.28* |
| Supportive care services | |  |  |  |  |  |  |
|  | Meals on wheels | 173 (50%) | 36.5 (43.9) | 204 (59%) | 44.0 (40.5) | -7.6 (-11.3; -3.8) | **0.67*** |
| Informal care | |  |  |  |  |  |  |
|  | Informal caregiver time | 156 (45%) | 139.5 (417.6) | 243 (70%) | 143.3 (349.7) | -3.7 (-45.1; 39.2) | 0.48* |

* p < 0.01

** Estimated using OECD data [17]

**Germany**

|  | | **RUD Lite**  (n=60) | | **InterRAI-HC**  (n=60) | | **Mean Difference**  **(RUD Lite minus interRAI-HC)** | **Spearman’s ρ** |
| --- | --- | --- | --- | --- | --- | --- | --- |
| *Service use category* | | Use of ser-vice, n (%) | Mean (SD) | Use of ser-vice, n (%) | Mean (SD) | Mean (95% CI) |  |
| Home care | |  |  |  |  |  |  |
|  | Home health and domestic care hours | 19 (32%) | 24.2 (53.1) | 55 (92%) | 63.8 (62.1) | -39.5 (-54.7; -24.7) | 0.38* |
| Physician visits | |  |  |  |  |  |  |
|  | Physician visits (GP + outpatient clinic visits | 60 (100%) | 2.8 (1.3) | 58 (97%) | 2.8 (1.7) | 0.1 (-0.3; 0.4) | **0.62*** |
|  | General practitioner visits | 60 (100%) | 2.6 (1.3) | - | - | - | - |
|  | Outpatient clinic visits | 12 (20%) | 0.3 (0.6) | - | - | - | - |
| Other health care services | |  |  |  |  |  |  |
|  | Physical therapy sessions | 14 (23%) | 2.9 (5.6) | 8 (13%) | 3 (9.1) | -0.2 (-2.9; 2.1) | 0.27* |
|  | Occupational therapy sessions | 6 (10%) | 1.1 (3.4) | 12 (20%) | 4.3 (11.1) | -3.3 (-6.5; -0.5) | 0.11 |
|  | Psychological treatment | 0.0 (0%) | 0.0 (0.0) | 0.0 (0%) | 0.0 (0.0) | - | - |
| Hospital admissions | |  |  |  |  |  |  |
|  | Hospital admission with overnight stay, times | 6 (10%) | 0.1 (0.3) | 4 (7%) | 0.1 (0.3) | 0 (-0.0; 0.1) | 0.13 |
|  | Hospital admission with overnight stay, nights | 6 (10%) | 1.2 (5.2) | 4 (7%) | 0.6 (2.3)** | 0.6 (-0.4; 2.0) | 0.14 |
|  | Nights general ward | 6 (10%) | 1.2 (5.2) | - | - | - | - |
|  | Nights ICU | 0 (0%) | 0.0 (0.0) | - | - | - | - |
|  | Emergency room visits without overnight stay | 0 (0%) | 0 (0) | 1 (2%) | 0.0 (0.3) | 0.0 (-0.1; -0.0) | 0 |
| Supportive care services | |  |  |  |  |  |  |
|  | Meals on wheels | 13 (22%) | 18.2 (35.4) | 18 (30%) | 25.8 (40.2) | -7.6 (-18.2; 1.5) | **0.59*** |
| Informal care | |  |  |  |  |  |  |
|  | Informal caregiver time | 52 (87%) | 375.2 (659.0) | 45 (75%) | 325.0 (508.0) | 50.2 (-27.4; 129.9) | **0.57*** |

* p < 0.01

** Estimated using OECD data [17]

**The Netherlands**

|  | | **RUD Lite**  (n=147) | | **InterRAI-HC**  (n=147) | | **Mean Difference**  **(RUD Lite minus interRAI-HC)** | **Spearman’s ρ** |
| --- | --- | --- | --- | --- | --- | --- | --- |
| *Service use category* | | Use of ser-vice, n (%) | Mean (SD) | Use of ser-vice, n (%) | Mean (SD) | Mean (95% CI) |  |
| Home care | |  |  |  |  |  |  |
|  | Home health and domestic care hours | 143 (97%) | 86.1 (67.9) | 141 (96%) | 81 (78.2) | 5.2 (-3.4; 13.8) | **0.80*** |
| Physician visits | |  |  |  |  |  |  |
|  | Physician visits (GP + outpatient clinic visits | 125 (85%) | 3.4 (4.1) | 101 (69%) | 2.6 (3.7) | 0.8 (0.4; 1.3) | **0.75*** |
|  | General practitioner visits | 101 (69%) | 1.8 (2.1) | - | - | - | - |
|  | Outpatient clinic visits | 74 (50%) | 1.7 (3.3) | - | - | - | - |
| Other health care services | |  |  |  |  |  |  |
|  | Physical therapy sessions | 58 (39%) | 5.7 (9.2) | 44 (30%) | 5.5 (9.4) | 0.2 (-0.6; 1) | **0.82*** |
|  | Occupational therapy sessions | 15 (10%) | 0.3 (0.9) | 1 (1%) | 0.2 (2.1) | 0.1 (-0.3; 0.3) | 0.27* |
|  | Psychological treatment | 3 (2%) | 0.1 (0.4) | 4 (3%) | 0.4 (2.1) | -0.3 (-0.6; -0.1) | **0.86*** |
| Hospital admissions | |  |  |  |  |  |  |
|  | Hospital admission with overnight stay, times | 13 (9%) | 0.1 (0.4) | 13 (9%) | 0.5 (2.1) | -0.4 (-0.7; -0.1) | **1.00*** |
|  | Hospital admission with overnight stay, nights | 13 (9%) | 0.5 (2.1) | 13 (9%) | 2.6 (11.0) | -2.1 (-3.8; -0.8) | **1.00*** |
|  | Nights general ward | 11 (7%) | 0.5 (2.1) | - | - | - | - |
|  | Nights ICU | 3 (2%) | 0 (0.2) | - | - | - | - |
|  | Emergency room visits without overnight stay | 13 (9%) | 0.1 (0.4) | 13 (9%) | 0.1 (0.3) | 0.0 (-0.0; 0.1) | **0.74*** |
| Supportive care services | |  |  |  |  |  |  |
|  | Meals on wheels | 24 (16%) | 8.2 (22.3) | 32 (22%) | 14.7 (30.4) | -6.5 (-10.3; -3.1) | **0.81*** |
| Informal care | |  |  |  |  |  |  |
|  | Informal caregiver time | 109 (74%) | 328.7 (664) | 103 (70%) | 312.2 (535) | 16.5 (-46.9; 85.5) | **0.70*** |

* p < 0.01

** Estimated using OECD data [17]
