# Supplementary material for: Convergent validity of the interRAI-HC for societal costs estimates in comparison with the RUD Lite instrument in community dwelling older adults
Source: BMC Health Serv Res. 2016 Aug 25;16(1):440. doi: 10.1186/s12913-016-1702-1 (PMC5000456; doi:10.1186/s12913-016-1702-1)
Supplement: Additional file 2: — Country specific cost of care estimates. Comparison of cost of care estimates (€) over a three month period between care utilisation assessed with the RUD Lite and interRAI-HC by country. (DOCX 28 kb) [file 12913_2016_1702_MOESM2_ESM.docx]

**Additional file 2.** Country specific cost of care estimates

Comparison of cost of care estimates (€) over a three month period between care utilisation assessed with the RUD Lite and interRAI-HC by country.

**Belgium**

|  | **RUD Lite**  (n=32) | **InterRAI-HC**  (n=32) | **Mean Difference**  **(RUD Lite minus interRAI-HC)** | **Spearman’s ρ** |
| --- | --- | --- | --- | --- |
| *Service use category* | Mean (SD) | Mean (SD) | Mean (95% CI) |  |
| Home health and domestic care | 6524 (5198) | 4871 (3659) | 1653 (680; 2768) | **0.81^*^** |
| Physician visits | 268 (500) | 203 (284) | 65 (-88; 271) | 0.06 |
| Other health care services | 682 (802) | 653 (944) | 30 (-230; 287) | **0.54^*^** |
| Hospital admissions | 946 (2738) | 836 (4700) | 109 (-1843; 1643) | 0.48^*^ |
| Supportive care services | 103 (213) | 98 (219) | 6 (-20; 43) | **0.92^*^** |
| Total medical care | 8523 (6054) | 6697 (5895) | 1826 (-480; 3902) | **0.60^*^** |
| Informal care | 14990 (28410) | - | - | **-** |
| Total societal costs | - | - | - | **-** |

* p < 0.01

**Finland**

|  | **RUD Lite**  (n=346) | **InterRAI-HC**  (n=346) | **Mean Difference**  **(RUD Lite minus interRAI-HC)** | **Spearman’s ρ** |
| --- | --- | --- | --- | --- |
| *Service use category* | Mean (SD) | Mean (SD) | Mean (95% CI) |  |
| Home health and domestic care | 2439 (3230) | 2716 (2463) | -277 (-546; 23) | 0.48* |
| Physician visits | 34 (70) | 38 (92) | -4 (-14; 5) | 0.36* |
| Other health care services | 22 (86) | 24 (117) | -2 (-14; 9) | 0.46* |
| Hospital admissions | 779 (3013) | 1502 (3580) | -723 (-1120; -326) | 0.46* |
| Supportive care services | 258 (310) | 311 (286) | -53 (-80; -26) | **0.67*** |
| Informal care | 1893 (5667) | 1944 (4746) | -51 (-612; 532) | 0.48* |
| Total societal costs | 5424 (8088) | 6535 (6340) | -1111 (-1854; -314) | **0.51*** |

* p < 0.01

**Germany**

|  | **RUD Lite**  (n=60) | **InterRAI-HC**  (n=60) | **Mean Difference**  **(RUD Lite minus interRAI-HC)** | **Spearman’s ρ** |
| --- | --- | --- | --- | --- |
| *Service use category* | Mean (SD) | Mean (SD) | Mean (95% CI) |  |
| Home health and domestic care | 920 (2018) | 2423 (2359) | -1502 (-2079; -938) | 0.37* |
| Physician visits | 98 (54) | 216 (129) | -118 (-148; -89) | 0.47* |
| Other health care services | 137 (266) | 222 (433) | -85 (-207; 24) | 0.35* |
| Hospital admissions | 612 (2571) | 310 (1169) | 302 (-240; 1032) | 0.14 |
| Supportive care services | 128 (250) | 182 (284) | -54 (-107; 0) | **0.59*** |
| Informal care | 5092 (8943) | 4410 (6894) | 682 (-371; 1763) | **0.57*** |
| Total societal costs | 6988 (9289) | 7763 (7471) | -775 (-2450; 966) | **0.52*** |

* p < 0.01

**Iceland**

|  | **RUD Lite**  (n=103) | **InterRAI-HC**  (n=103) | **Mean Difference**  **(RUD Lite minus interRAI-HC)** | **Spearman’s ρ** |
| --- | --- | --- | --- | --- |
| *Service use category* | Mean (SD) | Mean (SD) | Mean (95% CI) |  |
| Home health and domestic care | 1689 (1561) | 1648 (1616) | 41 (-93; 178) | **0.76*** |
| Physician visits | 96 (101) | 45 (91) | 51 (31; 71) | 0.40* |
| Other health care services | 205 (374) | 187 (390) | 18 (-38; 73) | **0.68*** |
| Hospital admissions | 916 (2352) | 551 (1208) | 365 (38; 745) | **0.56*** |
| Supportive care services | 105 (214) | 115 (222) | -10 (-42; 21) | **0.74*** |
| Informal care | 2629 (3655) | 3109 (5372) | -480 (-1497; 373) | **0.80*** |
| Total societal costs | 5641 (4839) | 5655 (6134) | -15 (-1026; 885) | **0.74*** |

* p < 0.01

**The Netherlands**

|  | **RUD Lite**  (n=147) | **InterRAI-HC**  (n=147) | **Mean Difference**  **(RUD Lite minus interRAI-HC)** | **Spearman’s ρ** |
| --- | --- | --- | --- | --- |
| *Service use category* | Mean (SD) | Mean (SD) | Mean (95% CI) |  |
| Home health and domestic care | 3274 (2581) | 3077 (2972) | 196 (-130; 526) | **0.80*** |
| Physician visits | 183 (275) | 206 (286) | -22 (-59; 19) | **0.72*** |
| Other health care services | 233 (363) | 249 (433) | -16 (-64; 28) | **0.79*** |
| Hospital admissions | 312 (1179) | 1296 (5472) | -984 (-1818; -316) | **0.91*** |
| Supportive care services | 58 (158) | 104 (214) | -46 (-73; -22) | **0.81*** |
| Informal care | 4461 (9010) | 4237 (7260) | 224 (-636; 1161) | **0.70*** |
| Total societal costs | 8520 (9492) | 9168 (9849) | -648 (-1898; 595) | **0.79*** |

* p < 0.01
